# Dynamic splinting for knee flexion contracture following total knee arthroplasty: a case report

**DOI:** 10.1186/1757-1626-1-421

**Published:** 2008-12-29

**Authors:** Eric Finger, F Buck Willis

**Affiliations:** 1 Physical Therapy, Sports medicine, Seton Southwest Hospital, Austin Texas, USA; 2 Health Physical Education, Recreation. Texas State University, San Marcos Texas, USA; 3 Clinical Research, Dynasplint Systems, Inc., Severna Park, Maryland, USA

## Abstract

Total Knee Arthroplasty operations are increasing in frequency, and knee flexion contracture is a common pathology, both pre-existing and post-operative. A 61-year-old male presented with knee flexion contracture following a total knee arthroplasty. Physical therapy alone did not fully reduce the contracture and dynamic splinting was then prescribed for daily low-load, prolonged-duration stretch. After 28 physical therapy sessions, the active range of motion improved from -20° to -12° (stiff knee still lacking full extension), and after eight additional weeks with nightly wear of dynamic splint, the patient regained full knee extension, (active extension improved from -12° to 0°).

## Introduction

Total Knee Arthroplasty (TKA) operations are increasing in frequency, from 160,000 operations in 2003, to an estimate of 500,000 per year by 2030 [1,2]. Knee flexion contracture is a common pathology following TKA [1-14], affecting up to 61% of these patients [3].

Contracture is defined as the shortening of the connective tissue [4,5] thereby stiffening the joint. The cause of flexion contracture following TKA operations has been suspected to arise from different hardware types [6] to pre-existing contracture prior to TKA [12]. Research has not proven a conclusive cause to the post TKA contracture, but the common opinion of surgeons is that flexion contracture is due to tightening of the posterior capsule combined with the tightening of biceps femoris and collateral ligaments [7].

Oullet and Moffet [8] examined this range of motion (ROM) deficit, and the effect it has on gait patterns. Gait lab analysis of their patients was performed less than one month post TKA and again following two subsequent months in therapy. They showed that while intensive therapy benefited the patients, the gait patterns were still impaired after two months. Their conclusion was that rehabilitation programs of greater intensity (increased frequency, intensity, or duration) should be undertaken soon after the TKA. Optimal knee joint function is dependant on full knee flexion and extension.

Increasing the AROM following this operation is imperative to the patient's complete recovery [6,9,10]. Both increased time at end range (of motion) [11] and stretch-bracing [9] are suggested for before and after the surgical procedure, providing the most effective course of action for the prevention and reduction of knee flexion contracture following TKA. The dynamic splinting could achieve both objectives, stretching and increased time at total end range.

Dynamic splinting utilizes the biomechanical adaptation of keeping the joint at end-range to achieve a physiological change of molecular realignment to elongate the connective tissue [5,14,15]. This protocol of low-load, prolonged-duration stretch with dynamic tension continually reduces the contracture.

Cook et al [12] revealed how pre-operative care in a comprehensive "Joint Replacement Program" (JRP) could benefit 74 TKA patients. Patients began this program 1–2 weeks prior to the TKA which exposed the patient to the protocols in the comprehensive treatment program and performed a full battery of tests to evaluate the pure difference before and after the TKA with the JRP. After the TKA the JRP used "Aggressive Physical Therapy" and while the JRP was considered very successful, not all of their patients achieved full knee extension [12].

The purpose of the report was to describe the benefits of using dynamic splinting as an adjunct to physical therapy in reducing contracture and regaining full knee extension following a TKA.

## Case presentation

This patient was 61-year-old male (6'0", 200 lbs) who presented with knee flexion contracture following a total knee arthroplasty, and his pre-operative maximal active range of motion (AROM) was -5° from full extension; (informed consent was obtained from this patient.) The patient was active in amateur golf and fitness training but had a 30 year history of previous knee injuries, osteoarthritis, and four previous knee surgeries. Following the TKA his active range of motion (AROM) was -20° in extension but knee flexion was unimpaired. The patient was reportedly previously active in sports and fitness training and would be compliant to all procedures and modalities used in the clinic and as home therapy.

Physical therapy was begun as primary intervention for this patient, one month following the TKA, and the protocols and modalities used included the following: Galvanic Stimulation ×15' + ice pack, Kinesiotape for swelling reduction, interferential current-electrical stimulation -80–150 mhz ×20', and 300 mv muscle stimulator at home in Russian and galvanic stimulation modes 2–3 x/day for 2 weeks.

Manual therapy included myofascial release to quadriceps (anterior thigh release: 1 minute hold; repeated 3 times) massage (kneading or petrissage and stripping), and joint mobilization (after swelling subsided). The joint mobilization included Flexion Restriction (patient seated), posterior glide of tibia on femur-grade-3; oscillations with 30 second hold, repeated 5 times with patellar mobilization of inferior glides ×5 minutes. The Extension Restriction therapy (patient prone with patella off of table) included anterior glide of tibia on femur, grade-3 oscillations, and static hold ×10 seconds in 3 repetitions, with patellar mobilization superior glides ×5 minutes.

The exercise program consisted of a combination of ROM, closed and open kinetic chain strengthening exercises, and proprioceptive/balance exercises targeting the trunk and lower extremity musculature. ROM exercises included heel slides both in supine and sitting and AAROM using pulleys to emphasizing joint surface conditioning. Stretching in prone and supine positions was used to increase knee extension ROM. Other exercises included partial body weighted squats, stationary cycling, gait training, and aquatic exercise therapy.

The patient had almost perfect compliance and attendance (missing and rescheduling only 2 of 28 appointments), and it was surmised that compliance to using dynamic splinting as home therapy would be effective because the patient was motivated and eager to try this new modality that has not yet been adopted as standard of care following TKA.

**Figure 1:**
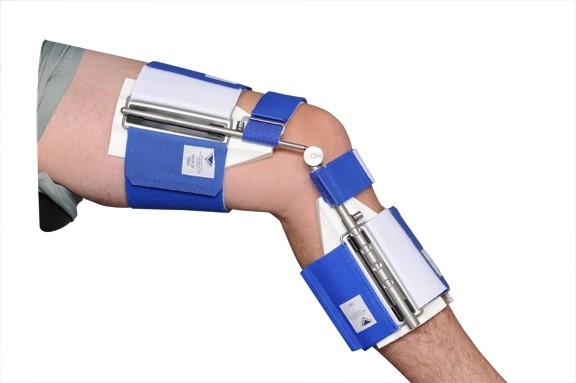
**Knee Extension Dynasplint**.

Dynamic splinting (DS) was used as a secondary intervention. After a course of 28 physical therapy sessions (twelve weeks), a Knee Extension Dynasplint (KED: Dynasplint Systems, Inc. Severna Park MD, USA) was prescribed for nightly wear to increase the patient's time at total end range of knee extension. (See Figure 1.) It was not prescribed initially because DS has not yet been established as standard of care following a TKA. The KED uses calibrated, replicable, bilateral, changeable tension technology to increased time at end range.

When the patient received the Dynasplint, a qualified consultant custom fit the unit and instructed the patient on how to don and doff the device. Verbal and written instructions were then provided for safety, general wear and care, and tension setting goals. The device is generally worn at night, while sleeping (6–8 hours), which would yield an additional 42–56 hours per week in home therapy.

After the patient spent the first week becoming accustomed to sleeping with the unit, the tension was then increased one increment every week, based on patient comfort. Increases in the tension setting result in increased torque values through the knee joint. Tension was increased from an initial setting at #1 (equalling 0.75 of a foot pounds of torque) to the #8 setting of tension (equalling 5.9 foot pounds of torque). If the patient experienced "post wear fatigue" following the use of the Dynasplint (soreness comparable to the feeling after a one hour session of aggressive physical therapy) for more than one hour, then he was then instructed to slightly reduce the time worn for the next two nights. However, the patient did not experience the "post wear fatigue" due to the gradual increases in tension.

After 28 physical therapy sessions, the active range of motion (AROM) in knee extension had progressed from a deficit of -20° to -12°. After the prescribed physical therapy was completed, the adjunct KED was prescribed. It was worn for six to eight hours per night for eight weeks, and the patient regained full AROM in knee extension, (0°). He reported mitigation of swelling and minimal pain with the return of functional activities, and soon returned to golfing, walking, and fitness training on a stationary bike.

## Discussion

The purpose of the report was to describe the benefits of using dynamic splinting as an adjunct to physical therapy in reducing contracture and regaining full knee extension following a TKA. The research by Denis et al proposed that additional stretching would be responsible for contracture reduction rather than continuous passive motion [10].

Ouellet and Moffett [8] stated that the most "Intensive rehabilitation programs (should occur) in the first months following TKA." Bellemans et al propose using "stretch-bracing" [9] in both the preoperative period (to reduce pre-existing contracture) as well as in the postoperative period to regain the full range of motion. Laskin and Beksac found problems with CPM equipment when used alone, but proposed using a knee splint for optimal biomechanical alignment in the "first few days after surgery" [11]. The additional 400 hours in home therapy with low-load, prolonged duration of stretching at end-range followed these recommendations in literature and benefited this patient's recovery.

## Conclusion

Before the TKA the patients had a maximal AROM of -5°. The patient presented with -20° AROM after the TKA; following physical therapy (two months) the AROM improved to -12° but still lacked full extension. After use of the Dynasplint for two additional months, the patient was discharged with full extension, (AROM of 0°). This allowed the patient to avoid a manipulation under anaesthetics to reduce the postoperative contracture.

## Abbreviations

AROM: Active Range of Motion; CPM: Continual passive motion equipment; DS: Dynamic splinting; KED: Knee Extension Dynasplint; TKA: Total Knee Arthroplasty; ROM: Range of motion.

## Competing interests

Mr. Finger has no competing interest. Dr Willis is employed by Dynasplint Systems Inc, but he has not ownership or stock options with this company.

## Authors' contributions

EF participated in the drafting of the manuscript, designed and implemented the rehabilitation program. BW participated in the drafting of the manuscript and extensive literature review. All authors read and approved the final manuscript.

## Consent

Written informed consent was obtained from the patient for publication of this case report and accompanying images. A copy of the written consent is available for review by the Editor-in-Chief of this journal.
